# The Effect of Probiotic Supplementation on Performance and the Histopathological Changes in Liver and Kidneys in Broiler Chickens Fed Diets with Aflatoxin B_1_

**DOI:** 10.3390/toxins11020112

**Published:** 2019-02-13

**Authors:** Katarzyna Śliżewska, Bożena Cukrowska, Stefania Smulikowska, Joanna Cielecka-Kuszyk

**Affiliations:** 1 Institute of Fermentation Technology and Microbiology, Department of Biotechnology and Food Science, Lodz University of Technology, 171/173 Wolczanska Street, 90-924 Lodz, Poland; 2 Department of Pathology, The Children’s Memorial Health Institute, 04-730 Warsaw, Poland; b.cukrowska@ipczd.pl (B.C.); j.kuszyk@ipczd.pl (J.C.-K.); 3 The Kielanowski Institute of Animal Physiology and Nutrition, Polish Academy of Sciences, 05-110 Jablonna, Poland; s.smulikowska@ifzz.pl

**Keywords:** aflatoxin B_1_, *Lactobacilli* and *Saccharomyces cerevisiae* probiotic, liver, kidney, performance

## Abstract

The aim of the study was to investigate the toxic effects of aflatoxin B_1_ (AFB_1_) and efficacy of a probiotic preparation containing *L. reuteri*, *L. plantarum*, *L. pentosus*, *L. rhamnosus* and *L. paracasei* and *Saccharomyces cerevisiae* yeasts to ameliorate their effects in broiler chickens. A total of 168 one-day-old female Ross 308 broilers were randomly allocated to six groups. Three wheat and soybean meal-based diets were prepared: Control diet and diets contaminated with 1 or 5 mg/kg AFB_1_ supplied in moldy wheat. All diets were unsupplemented or supplemented with probiotic, cold pelleted and fed from 1 to 35 day of life. Feeding diet with 1 mg AFB_1_/kg did not affect performance, but a diet with 5 mg AFB_1_ resulted in a significant reduction of feed intake and BWG, both diets induced liver and kidneys enlargement. The probiotic supplementation of the diets partially ameliorated those negative effects and resulted in a significant increase of AFB_1_ excretion. It was accompanied by the reduced level of AFB_1_ residues in the liver from 8.9 to 3.7 and from 11.8 to 5.9 µg/kg, in kidneys from 7.9 to 2.5 and from 13.7 to 4.1 µg/kg in birds fed the less and more contaminated diets, respectively. AFB_1_ exposure caused many severe histopathological changes in the liver and kidneys of broilers, probiotic supplementation significantly reduced the changes of these organs. It may be concluded that the probiotic supplementation can be used to alleviate the negative effects of contamination of broiler feed with AFB_1_ on bird health and product security.

## 1. Introduction

There are many biological toxins present in the natural environment, which may be dangerous for human and animal health. Mycotoxins are currently considered to be among the most dangerous ones. They are toxic secondary metabolites of some species of filamentous fungi, mainly belonging to the genera *Aspergillus*, *Penicillium* and *Fusarium* [1].

From the chemical point of view, mycotoxins constitute a heterogeneous group of compounds, belonging to over 20 groups. They are, among others, derivatives of coumarin (aflatoxins, ochratoxins), polycyclin lactones (zearalenone, patulin), derivatives of trichothecenes (toxin T-2, nivalenol, deoxynivalenol) [2]. Among 18 types of aflatoxins identified, aflatoxin B_1_ (AFB_1_) is the most toxic and the most common one, followed by aflatoxins G_1_, B_2_ and G_2_, which demonstrate lesser toxicity [3,4].

Aflatoxins are carcinogenic contaminants of food and feed that are frequently responsible for health and economic problems in many countries [5]. High-dose exposure to aflatoxin may cause vomiting, abdominal pain, and even death, while chronic exposure to small quantities may lead to liver cancer [6]. The International Agency for Research on Cancer (IARC) has classified both B- and G-type aflatoxins as Group 1 mutagens [7,8].

None of the geographic regions is free from mycotoxins. The data published by FAO in 2001 showed that 25% of agricultural raw materials were contaminated with mycotoxins, and their type and contamination levels were greatly dependent on the climatic zone. The conditions for the synthesis of mycotoxins in Europe are less favorable than in North America or Asia, but the problem of the presence of mycotoxins in grains is very important also for numerous European countries. Therefore, contamination of animal feeds with mycotoxins is considered to be a world-wide problem [9,10].

The presence of aflatoxins—mostly of AFB_1_—was confirmed in numerous products, including cereal grains, soya beans, corn, sorghum, oilseed meals [11]. The current European Union levels for AFB_1_ are 2 ppb for food and 20 ppb for feed [12]. Many countries, including the EU states, implemented measures aimed at the reduction of aflatoxins content in food and feed products.

Contaminated plant products have to be decontaminated. Biological decontamination of products may be carried out both in vitro—by removal of the toxin from a feed, before giving it to an animal, or in vivo—in animal’s alimentary tract [13,14]. Detoxification may be a result of metabolic transformation of mycotoxins by microbiota or may occur through their adsorption to bacteria or yeasts cell walls. Biological methods involve not only viable and non-viable microorganisms, but also fragments of their cells, such as fragments of yeast cellular walls, or even their proteins. Among others, lactic acid bacteria (LAB) and *Saccharomyces cerievisiae* cells are known to bind different molecules or complex structures, among others mycotoxins, on the cell wall surface [14]. Residues of aflatoxins may exist in the tissues of animals receiving contaminated feeds becoming a potential human health hazard. Application of LAB to animal diets has been considered as appropriate for the detoxification of aflatoxins [13], however, available literature concerning broiler chickens remains scanty.

The aim of the study was to evaluate the toxic effects of AFB_1_-contaminated diets and efficacy of a probiotic preparation composed of LAB and *Saccharomyces cerevisiae* yeasts to reduce their effects on broiler chickens growth performance, health status and aflatoxin residue concentration in the liver and kidneys.

## 2. Results

### 2.1. Performance Indices

No mortality was recorded among control birds and birds that were fed diets with 1 mg AFB_1_/kg, but during the last week of the experiment 2 birds died in the group fed the diet with 5 mg AFB_1_/kg and 1 bird in the group fed the diet with 5 mg AFB_1_/kg supplemented with probiotic (Table 1). Feeding chickens for 35 days with diet contaminated with 1 mg AFB_1_/kg had no significant effect on body weight gain (BWG), feed intake and feed conversion ratio (FCR), compared to chickens fed the control diet (Table 1). The addition of the probiotic to control diet and to the diet contaminated with 1 mg AFB_1_/kg did not affect performance indices. Only a high contamination level (5 mg AFB_1_/kg diet) resulted in a significant reduction of feed intake and BWG. The probiotic supplementation of the diet containing 5 mg AFB_1_ improved feed intake and BWG significantly, but not to the level obtained in birds fed control and the less contaminated diets.

Feeding chickens with both AFB_1_ contaminated diet, increased the weight of liver and kidneys significantly, compared to the control group (Table 2). The addition of the probiotic to the control diet did not affect the weight of liver and kidneys, the probiotic added to the diet with 1 mg AFB_1_/kg contributed to the restoration of a weight of liver and kidneys to a similar as in control group. The probiotic added to the diet with a high level of AFB_1_ reduced the weight of liver and kidneys significantly but still they remained enlarged in comparison with other groups.

### 2.2. The Concentration of Aflatoxin B_1_ in Excreta

**Figure 1:**
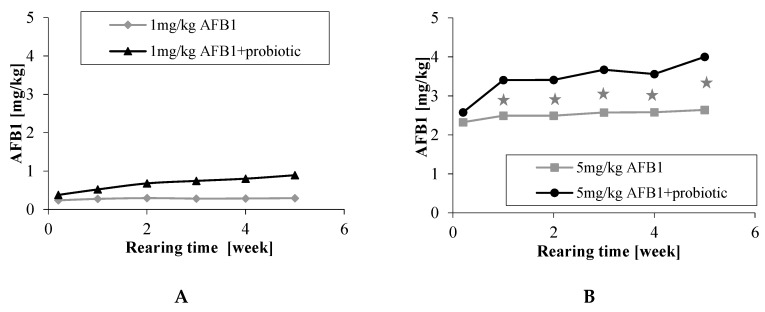
Concentration of aflatoxin B_1_ in excreta during rearing, *n* = 14–16 (see Table 1): (**A**) chickens fed diets with 1 mg AFB_1_/kg; (**B**) chickens fed diets with 5 mg AFB_1_/kg; 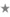 differences between groups significant at *p* < 0.05.

Aflatoxin B_1_ was not detected in excreta of chickens fed control diet without or with probiotic. The concentration of AFB_1_ in excreta in chickens fed contaminated diets depended on its dietary concentration but throughout the whole period of the experiment was higher in chickens consuming diets supplemented with probiotic (Figure 1). At the end of the experiment the concentration of AFB_1_ in excreta of chickens eating diets contaminated with AFB_1_ at a level of 1 and 5 mg/kg was, on average, 0.29 and 2.74 μg/kg, respectively, but was 3-fold and by 46% higher (*p* < 0.05) in chickens provided respective diets supplemented with probiotic (Table 3).

### 2.3. Concentration of Aflatoxin B_1_ in Liver and Kidneys

Aflatoxin B_1_ was not found in livers and kidneys of chickens fed control diet without or with probiotic. The mean concentration of AFB_1_ in the liver tissue of chickens consuming diet contaminated with AFB_1_ at a level of 1 and 5 mg/kg was 8.86 and 11.84 µg/kg, respectively. In chickens eating respective diets supplemented with the probiotic the concentration of AFB_1_ in the liver was, on average, by 58% and 50% lower as compared to chickens fed the diets without probiotic (Table 3). Differences between unsupplemented and supplemented groups are statistically significant (*p* < 0.05).

In chickens receiving the diet with 1 and 5 mg/kg AFB_1_ the mean concentration of AFB_1_ in kidney tissue was 7.93 and 13.71, respectively, whereas in chickens obtaining those diets supplemented with the probiotic it was, on average, by 68% and 70% lower (Table 3). Differences between unsupplemented and supplemented groups were statistically significant (*p* < 0.05).

### 2.4. Histopathological Changes in Liver and Kidneys

**Figure 2:**
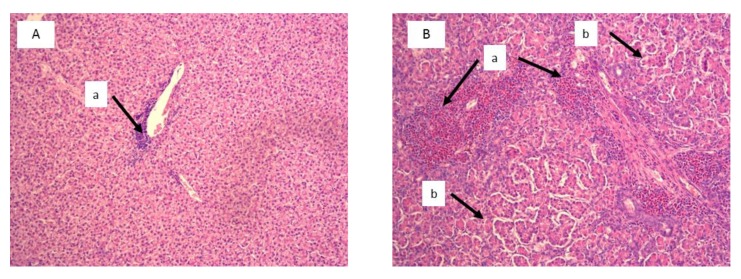
Liver histology of selected chicken form the control group (**A**) and from the group fed with the diet contaminated with 5 mg/kg AFB_1_ (**B**). Visible discrete (Aa) and increased (Ba) eosinophilic infiltrations, and signs of micronodular cirrhosis with increased division of the hepatic parenchyma into fine nodules (Bb) (H&E staining, magnification × 100).

**Figure 3:**
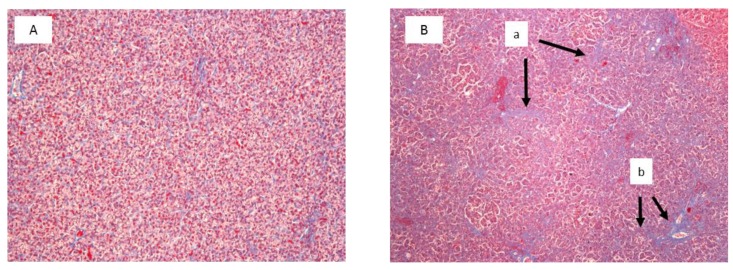
Azan staining of liver specimens of the selected control chicken (**A**) and the chicken fed with the diet contaminated with 5 mg/kg AFB_1_ (**B**). Visible growth of fibrous tissue in the hepatic parenchyma (a) and fibrosis of portal spaces with fibrous bridges (b) (Magnification × 100).

**Figure 4:**
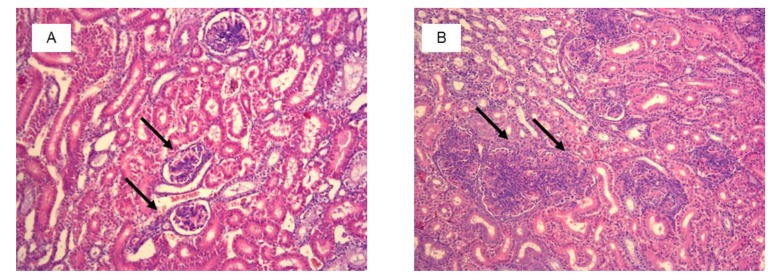
Kidney histology of selected chicken from the control group (**A**) and from the group fed with the diet contaminated with 5 mg/kg AFB_1_ (**B**). Arrows show normal glomeruli of 100–150 µm diameter in control chicken (**A**) and enlarged glomeruli of 200–400 µm diameter with increased number of cells and mesangial matrix in AFB_1_ chicken (**B**) (H&E staining, magnification × 100).

**Figure 5:**
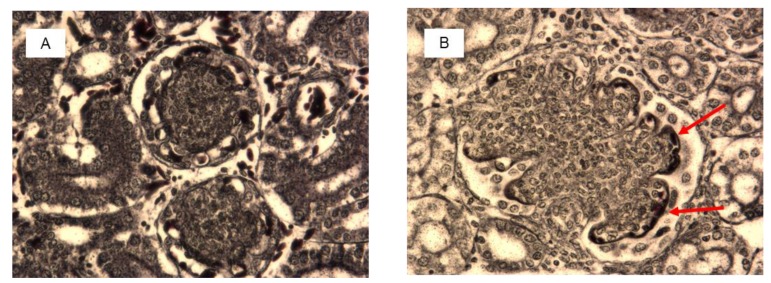
Wilder staining of kidney specimens of the selected control chicken (**A**) and the chicken fed with the diet contaminated with 5 mg/kg AFB_1_ (**B**). Red arrows show thickening of the basal membrane in glomeruli of AFB_1_ chicken (Magnification × 400).

Histological examination showed significant damage in the liver of chickens that were fed the diets contaminated with AFB_1_ at both concentrations (Table 4), whereas lesions in kidneys were found only in chickens consuming the diet with 5 mg AFB_1_/kg. Livers of chickens that were fed the diet with 1 mg of AFB_1_ presented a slight infiltration of perilobular eosinophilic cells, and a slight to moderate vascular degeneration and fatty changes of hepatocytes. In chickens that were fed a diet with 5 mg of AFB_1_ the infiltration of eosinophilic cells was more intense and occurred both in peri- and interlobular areas. A division of the hepatic parenchyma into fine nodules was also observed as signs of micronodular cirrhosis (Figure 2). In addition to predominantly moderate vascular degeneration and fatty changes of hepatocytes, moderate hyperplasia of the bile tract epithelium and intensive fibrosis were found in livers of those chickens (Figure 3). In the kidneys, histological changes involved enlargement of the renal glomeruli and an increase of the mezangium matrix (Figure 4). Wilder staining revealed also thickening of the basal membrane in the glomeruli (Figure 5).

The probiotic supplementation of the diet contaminated with 5 mg AFB_1_/kg reduced histopathological changes in kidneys, whereas changes in the liver were fully reduced only at a lower concentration of AFB_1_ in the diet. At a higher dose of AFB_1_ the probiotic failed to prevent histological changes in the liver, but accounted for the reduction of their intensity.

## 3. Discussion

Biological elimination of mycotoxins from food and feed and lowering their negative effects in a human and animal organisms is a novel and a very promising method. Among organisms used in research on elimination of mycotoxins, one should mention *Acinetobacter calcoaceticus* [15], *Bifidobacterium* sp. [16,17], *Enterococcus faecium* [18], *Flavobacterium aurantiacum* [19], *Lactobacillus* sp. [20], *Bacillus subtilis* [21], *Pseudomonas* sp. [22], *Rhodococcus erythropolis* [23], and also moulds belonging to the *Rhizopus* [24] and *Aspergillus* [25] geni, as well as *Saccharomyces* yeast [26].

The mechanism of removing mycotoxins from the environment by microorganisms has not been thoroughly explained so far. It arises from the available literature that in the case of LAB and yeast, it is the result of cell wall adhesion, which is additionally confirmed by the fact that even dead cells preserve the said activity [27,28].

In the mechanism of mycotoxin adsorption to bacterial cells an important role may be played by peptidoglycans and exopolysaccharides in the cell, teichoic acids and the reactions related to cell hydrophobicity and electrostatic properties [29]. In the case of yeast, the most significant role is ascribed to β-D-glucans and mannan oligosaccharides of the cell wall [30]. Pizzolitto et al. [31] additionally stated that van der Waals forces, hydrogen bonds or hydrophobic interactions are also involved in the absorption process.

Probiotics composed with LAB should arouse the greatest interest, considering their beneficial, physiological properties in human and animal organism, among others in broiler chickens [13,32,33]. Moreover, the LAB are able to inhibit the growth of moulds, and the production of mycotoxins [34]. The present study evaluated the ability of probiotic preparation composed of LAB and *S. cerevisiae* yeasts to counteract the toxic effects of AFB_1_ in broiler chickens.

Probiotics are subject to the rules contained in the general food law, according to which they should be safe for animal and human health. In the USA, microorganisms used for oral intake should be classified as GRAS (generally regarded as safe), and be regulated by Food and Drug Administration. In Europe, the European Food Safety Authority (EFSA) introduced the term QPS (Qualified Presumption of Safety). The QPS concept takes into account additional criteria of the safety rating of bacterial additives, such as the history of safe use and the lack of risk becoming antibiotic-resistant. In the study, *Lactobacillus* bacteria and *Saccharomyces cerevisiae* yeast were used, classified as both GRAS and QPS and therefore safe for animal and human health.

In the present study feeding chickens for 35 days with the diet contaminated with 1 mg/kg of AFB_1_ had a minor effect on feed intake, BWG and FCR compared to birds fed with the control diet. The addition of the probiotic to that diet resulted in insignificant improvement of BWG by 4%, on average. Supplementation with probiotic of the control diet and the diet contaminated with 1 mg/kg AFB_1_ had no beneficial effect on the growth performance of broilers.

Rodricks and Stoloff [35] observed a reduction of BWG by 5–10% as a result of feeding chickens with the diet containing 2.5 mg AFB_1_/kg. Using the same dose of AFB_1_, Hamilton et al. [36] reported a reduction of BWG by 81 g, and with the dose of 5 mg AFB_1_/kg the reduction of BWG by 164 g, on average. Smith et al. [37] demonstrated that the administration of a mixture of aflatoxins (79% AFB_1_, 16% AFG_1_, 4% AFB_2_ and 1% AFG_2_) to chickens in the amount of 3.5 mg/kg diet resulted in reduced BWG, and an enlargement of liver and kidneys. An enlargement of liver and kidneys of chickens that were fed a diet contaminated with 5 mg/kg AFB_1_ was also reported by Pimpukdee et al. [38]. Quezada et al. [39] feeding chickens with the diet containing 2 mg/kg AFB_1_ obtained the increase of mass of kidneys at 21st day of age and 20–30% BWG reduction in 4-week-old chickens.

In the current study we have observed a reduction of BWG by 20–50% in chickens that were fed a diet with 5 mg of AFB_1_/kg and a significant increase in the relative weight of liver and kidneys on both AFB_1_ contamination levels compared to the control group. The relative weight of those organs in chicken that were fed a diet contaminated with 1 mg of AFB_1_/kg was 34 and 24% higher, though in chickens that were fed a diet with 5 mg of AFB_1_/kg it was 73% and 122% higher compared to control. Obtained results are therefore consistent with those reported by Smith et al. [37], Pimpukdee et al. [38], and Quezada et al. [39].

In the present study the probiotic added to the diet containing 1 mg AFB_1_/kg contributed to the restoration of the relative weight of liver and kidneys to similar as in the control group. The positive effect of probiotic supplementation was also observed in chickens fed the diet containing 5 mg AFB_1_/kg but both organs remain enlarged by 59% and 100% compared to the control birds.

The aflatoxins present in the feed might be absorbed, transformed by intestinal tract microbiota to less toxic metabolites, or excreted in droppings. In the present study the excreta of chickens fed unsupplemented diets contaminated with 1 and 5 mg AFB_1_/kg contained, on average, 0.29 and 2.74 mg AFB_1_/kg, respectively. The addition of the probiotic to the AFB_1_-contaminated feed contributed to a significant increase in the concentration of AFB_1_ in droppings. There is substantial evidence that LAB and *S. cerevisiae* yeasts, present in the probiotic preparation used in the experiment are capable of effective binding of toxins [13,14,21,26]. Moreover, it was evidenced by our in vitro study [40] that a probiotic composed with LAB and *S. cerevisiae* has been active in metabolic transformation of AFB_1_, as the concentration of toxin in broiler diets contaminated with 1 and 5 mg AFB_1_/kg decreased by 55 and 39%, respectively, after 6 h incubation. 

The main site of absorption of mycotoxins in animals is the small intestine (in birds mainly the proximal part of the jejunum). The absorption takes place as a result of both an active transport, and a passive diffusion and with blood in the portal vein absorbed mycotoxins reach the liver and other organs [41]. The greater part of absorbed mycotoxins is removed with urine (in birds excreted with droppings), but residues are accumulated in liver, kidneys and muscles and pose a threat for animal and human health [42].

In the present study the concentration of AFB_1_ in the liver tissue was 8.9 and 11.8 µg/kg; in kidneys 7.9 and 13.7 µg/kg in chickens that were fed diets with 1 and 5 mg AFB_1_/kg, respectively. Tejada-Castañeda et al. [43] reported, that after 21 days of feeding chickens with diet contaminated with 2 µg AFB_1_/kg the concentration of the toxin in the liver tissue was 1–1.2 µg/ kg and in the kidneys tissue it was 0.8 µg/kg. We cannot find reports concerning the protective effect of LAB probiotics on the AFB_1_ residues accumulation in broilers body tissues, but Fan et al. [21] reported, that *Bacillus subtilis* probiotic lowered AFB_1_ residues in the liver of broilers by 42–97%, depending on dose. In the present study the AFB_1_ concentration in the liver of broilers that were fed diets containing 1 and 5 mg AFB_1_/kg and supplemented with the probiotic was reduced by about 50% (to 3.7 and 5.9 µg/kg, respectively), in kidneys by about 70% (to 2.5 and 4 µg/kg, respectively) compared to the birds fed contaminated but non-supplemented diets. These results show that the LAB and *S. cerevisiae* probiotic used in the study AFB_1_ can be used against the occasional contamination of broiler feed with aflatoxins.

In the study we assessed changes in livers and kidneys, organs the most exposed to aflatoxin B_1_. The histopathological examination demonstrated the presence of kidney injuries only in chickens fed the diet contaminated with 5 mg AFB_1_/kg. Changes involved increased diameter of the glomeruli, an increased amount of the matrix and mesangial cells and a thickening of basal membranes of the glomeruli. The addition of the probiotic to the diet caused a reduction of the diameter of most of the glomeruli, even back to the normal value. Pathological changes were found in livers of chickens fed with both contaminated diets. They were: Eosinophil infiltrations, vascular degeneration and fatty changes of hepatocytes. Additionally, signs of micronodular cirrhosis with highly intensified eosinophilic infiltration were found in livers of chickens fed with the diet containing 5 mg AFB_1_/kg. Histological changes were not observed in livers and kidneys of chickens fed with the probiotic-supplemented diets containing 1 mg AFB_1_/kg and were of lesser intensity in chickens fed the probiotic-supplemented diet with 5 mg AFB_1_/kg. It confirmed the other reports that LAB and yeast from the probiotic are able to effectively bind AFB_1_ [33] and/or to transform it to a non-toxic compound [40]. Due to that less toxin has reach liver and in kidneys and a resulted histological changes were not as pronounced as it was in case of chickens receiving contaminated but non-supplemented diets.

According to literature, in chickens aflatoxins may cause degradation of proximal tubules, the formation of hyaline concrements in the free space of the Bowman capsule, atrophic glomeruli and cortical fibrosis [44]. Symptoms of acute liver damage include increased fragility of blood vessels, coagulopathy, hemorrhage and prolonged presence of blood clots. The color of livers turns from yellow to copper, the gallbladder becomes enlarged and bile gets thinner. Hypertrophy of the biliary tract ensues [45]. According to Solcan et al. [46] the size of mycotoxin-induced lesions in chicken liver and kidneys are dose-dependent. The authors noted minor degenerative changes in the liver and kidneys for the lowest dose, more pronounced ones for 5 and 9 mg OTA/kg, and for the dose of 20 mg/kg changes were defined as serious. Pizzolitto et al. [47] demonstrated that *Saccharomyces cerevisiae* CECT 1891 yeast, added to a diet (10^10^ cells/kg) or to drinking water (10^9^ cells/L) had a beneficial protective effect on histopathological changes of the liver of broiler chickens fed with the diet contaminated with 1.2 mg of AFB_1_/kg.

The aim of the study was to evaluate the aflatoxin B_1_ detoxification potential of the probiotic preparation composed with LAB and *S. cerevisiae* in the gastrointestinal tract of chickens. The results indicate that the probiotic preparation may therefore be used for prevention of mycotoxicoses in broiler chickens.

## 4. Conclusions

Addition of the probiotic preparation composed with LAB and *S. cerevisiae* to AFB_1_-contaminated diet has a positive effect on performance, reduces AFB_1_ residues concentration and prevents or reduces degenerative changes in the liver and kidneys. As a consequence the probiotic preparation may be used in the prevention of mycotoxicoses in chicken, contributing to the improvement of animal health, and the quality of meat.

## 5. Materials and Methods

### 5.1. Probiotic Preparation

The probiotic preparation used contained (per 1 kg): 4.5 × 10^10^ *Lactobacillus* cells (*L. reuteri* LOCK 1092, *L. plantarum* LOCK 0860, *L. pentosus* LOCK 1094, *L. rhamnosus* LOCK 1091, *L. paracasei* LOCK 1091) and 4 × 10^6^ yeast *Saccharomyces cerevisiae* LOCK 0119 cells. The strains derived from the Centre of Industrial Microorganisms (LOCK), Institute of Fermentation Technology and Microbiology, Lodz University of Technology in Poland. The preparation possesses full probiotic documentation and is licensed [48].

### 5.2. Preparation of Diets

Wheat grain was inoculated with the strain of *Aspergillus flavus* K30 and incubated to obtain high concentration of aflatoxin B_1_ in accordance with a procedure described by Xiao et al., [47]. Twenty Erlenmeyer flasks (500 mL), each containing 30 g of feed-grade wheat and 30 mL of distilled water, were autoclaved for 30 min at 120 °C and then inoculated with *Aspergillus flavus* K30. The culture was maintained at 30 °C in a dark room. The fermentation was terminated on day 12 of incubation, the contaminated wheat was dried for 72 h, ground, and the aflatoxin B_1_ concentration was determined.

Experimental diets were prepared, each covered satisfactorily the requirements suggested by the NRC [49] for broiler chickens. The composition of control diets are shown in Table 5, experimental diets were prepared to meet the final concentration of 1 or 5 mg of aflatoxin B_1_ per kg of diet by substituting respective amounts of contaminated wheat by feed-grade wheat. Diets were prepared without or with probiotic preparation. All ingredients were thoroughly mixed and cold pelleted with the use of CL-2 CPM laboratory pellet mill (California Pellet Mill Co., Crawfordsville, IN, USA).

### 5.3. Experimental Design, Birds and Sample Collection

A total of 168 one-day-old male Ross broiler chicks were used. They were randomly assigned to six groups and housed in electrically controlled breeder (4 cages × 7 chickens per group) for the first week of life. On the 8th day of life, after 4 h of feed deprivation chickens were weighed, 16 birds with a body weight close to the group average were selected in each group and housed in individual metabolic cages in environmentally controlled room. A 3 × 2 factorial arrangement was used, the factors being dietary AFB_1_ concentration (0, 1 and 5 mg per kg) and without or with probiotic (Table 6). Chickens were fed with Starter-type diets for the first 14 days of experiment, from day 15 to 28 with Grower-type diet, and during the last seven days with Finisher-type diet (Table 5). The experimental protocol was approved by the Local Animal Care and Use Committee in Warsaw, Warsaw University of Life Sciences, Poland (1/2016, date of approval: 12 January 2016).

Bird weight and feed consumption was recorded weekly, mortality was registered as it happens. Excreta were collected during the last day of each week, homogenized and 10 g samples were frozen for AFB_1_ determination. After the last weighing on day 35 of life, birds were killed by cervical dislocation. Liver, kidneys and the alimentary tract filled with content were excised and weighed. Feed consumption, body weight gain, feed conversion ratio as well as the relative weight of liver and kidneys were calculated. Liver and kidneys tissues were sampled from 10 chickens in each group for AFB_1_ determination and histopathological assessment.

### 5.4. Aflatoxin B_1_ Concentration

The AFB_1_ concentration was determined in excreta (16 samples in a group at each week of the experiment) and in the liver and kidneys with an enzyme linked immunosorbent assay (ELISA) and high-performance liquid chromatography (HPLC).

#### 5.4.1. Reagents

All chemicals were analytical reagent grade, excepting methanol and acetonitrile (HPLC grade). They were purchased from Merck (Darmstadt, Germany). All solutions were prepared with deionized water. Standard of AFB_1_ was purchased from Sigma Chemical Co. (St. Louis, MO, USA). AFB_1_ standard stock solution was prepared in benzene-acetonitrile (98:2, *v*/*v*).

#### 5.4.2. ELISA Method

Commercial ELISA kit AgraQuant Aflatoxin (Romer Labs Diagnostic, Singapore) was used in the study. These ELISA assay kit is a direct competitive immunoassay with horseradish peroxidase conjugate. The assays were performed according to the procedure described in the AgraQuant Assay kit manual. The AFB_1_ was extracted by mixing 10 g of sample (excreta, liver or kidneys) with 50 mL methanol:water (70:30, *v*/*v*), the mixture was filtered. The 100 μL of horseradish peroxidase conjugate solution and 50 μL of filtrate or standard AFB_1_ solution was added to the well and mixed thoroughly. Then 50 μL of the mixture were transferred to well containing antibodies and incubated for 15 min at room temperature. After incubation, wells were rinsed five times with distilled water and dried. The 50 μL of substrate (urea peroxide) was added to each well, after 5 min at room temperature the reaction was stopped by adding 50 μL of stopping solution (1M sulfuric acid). The absorbance of the solution was read with a UVM 340 microplate reader (Asys, Eugendorf, Austria) at a wavelength *λ* = 450 nm, and calculated by extrapolating the OD with the respective AFB_1_ calibration curve [50].

#### 5.4.3. HPLC Method

The samples (excreta, liver or kidneys) were analyzed for the presence of AFB_1_ using an immunoaffinity column for clean up and HPLC with fluorescence detection. Using 10 g of sample, aflatoxin extraction was done with 50 mL methanol:water (70:30, *v*/*v*). The samples were centrifuged at 1600× *g* for 10 min and the upper fat layer was discarded. Then, 50 mL was passed through an immunoaffinity column (AflaStar, Romer Labs Diagnostic, Singapore). The column was washed with 25 mL of PBS buffer (pH 7.4) to remove extraneous non-specific material. The AFB_1_ bound to the antibody was released by the elution with 2.0 mL methanol. The eluate was evaporated to dryness using an N_2_ stream. The residue was dissolved in 400 µL mobile phase and a 20 µL aliquot was injected into the HPLC equipment.

HPLC was performed on Waters system (Waters 600, Waters, Milford, MA, USA) equipped with a Quat Pump, a fluorescence detector at wavelengths of 360 and 430 nm for excitation and emission, respectively. The HPLC column was a LiChrosorb C18 (250 × 4 mm with 5 µm granulation, Merck, Darmstadt, Germany) and guard column Phenomenex C18, 4 × 3 mm. The mobile phase consisted of acetic acid 2% aqueous solution–acetonitrile–methanol (60:30:10; *v*/*v*/*v*) and the flow rate was 1 mL/min [51].

### 5.5. Histopathological Examination

Specimens collected from the liver and the kidney were fixed in 4% buffered formalin (Chempur, Piekary Śląskie, Poland) for 24 h at room temperature. The material in histopathological cases (Leica, Wetzlar, Germany) was placed in a tissue processor TP 1020-1 (Leica, Wetzlar, Germany), where it was passed through a series of increasing concentrations of alcohol (70%, 90%, 96%, 99.8%), xylene and paraffin. Then the material was embedded in paraffin, using the corresponding equipment EG-1160 (Leica, Wetzlar, Germany). The embedded material was sliced into 4-µm specimens using a metal knife (Shandon MX35, ThermoFisher Scientific, Waltham, MA, USA) with a microtome RM 2265 (Leica, Wetzlar, Germany). Specimens were placed on syalinised slides (Surgipath, Leica Biosystem, Wetzlar, Germany). Slides with specimens were then placed in an incubator at 60 °C for 24 h, and then de-paraffined. The material was de-paraffined with xylene and decreasing concentrations of ethanol (99.8%, 96%, 50%). The material was placed in each of the reagents for 3 min. Specimens were hematoxylin and eosin stained (H&E staining) for 1–2 min and closed with glycerogel (Mounting Medium, DakoCytomation, Glostrup, Denmark). Sections obtained from each organ were examined under the Olympus light microscope at magnifications ×100 (H&E and Azan staining), and ×400 (Wilder staining). Specimens of the liver were examined for previously described lesions: vascular changes and fatty degeneration of hepatocytes, inter-and perilobular inflammation, bile duct hyperplasia/hypertrophy and fibrosis. Sections with no, mild, moderate, or severe lesions were given scores of 0, 1, 2, and 3, respectively.

#### 5.5.1. Azan Staining of Specimens

After de-paraffination, specimens were incubated for 7 min with 1% aniline alcohol (a solution of aniline and 96% ethanol), and then washed with 96% ethanol and running water, for 3 min. After washing specimens were incubated at 56 °C for 20 min in azocarmine G solution (0.1 g of azocarmine G, 100 mL of distilled water, 1 mL of acetic acid). After incubation, specimens were washed in distilled water and washed with 1% aniline alcohol solution and with acidic alcohol (a solution containing 1 mL of acetic acid and 99 mL of ethanol). Then, specimens were incubated at room temperature for 10 min with 10% phosphotungstic acid. After washing specimens with distilled water, they were incubated for 3 min with the Mallory’s mixture (0.5 g of aniline blue, 2 g of yellow orange G, 100 mL of distilled water, 8 mL of glacial acetic acid). Specimens closed with glycerogel (Mounting Medium, DakoCytomation, Glostrup, Denmark) were examined under the Olympus light microscope (Olympus, Tokyo, Japan).

#### 5.5.2. Wilder Staining of Specimens

De-paraffined specimens were incubated in 5% periodic acid for 15 min, and then in 5% oxalic acid for 2 min. After washing with distilled water, specimens were incubated for 2 min with 3% H_2_O_2_ solution. Specimens, washed with tap water (3 min) and distilled water, were impregnated with ammonia silver for 7 min. Ammonia silver was obtained by combining 5 mL of 10% silver nitrate AgNO_3_ with 5 mL of 3% NaOH, and ammonia added dropwise until dissolution of the formed sediment. The prepared solution was diluted with equal volume of distilled water. After washing with 96% ethanol, specimens were incubated for 2 min with 10% formalin solution, washed with distilled water and immersed in 1% gold chloride solution for 2 min. After washing specimens with distilled water, they were incubated for 2 min with 5% sodium thiosulphate solution, and then washed with distilled water. Specimens closed with glycerogel were examined under the Olympus light microscope.

### 5.6. Statistical Analysis

The data were analyzed using the STATISTICA 8.0 software package (Statsoft Co., Krakow, Poland). One-way analysis of variance (ANOVA) was applied to assess the effects of diets on the concentration of aflatoxin B_1_ in excreta, liver, kidneys and on performance indices. If the analysis revealed a significant influence (*p* ≤ 0.05), the differences among the groups were analyzed with Duncan’s multiple range post hoc test (*p* ≤ 0.05).

## Figures and Tables

**Table 1:** Performance indices ^1,2^.

Time of Rearing	Experimental Group					
	1 AFB_1_	1 AFB_1_ + Probiotic	5 AFB_1_	5 AFB_1_ + Probiotic	Control	Control + Probiotic
Body weight gain (BWG), g						
1 week	123 ± 1.51 ^b^	125 ± 1.34 ^b^	95 ± 2.21 ^a^	98 ± 1.85 ^a^	134 ± 3.14 ^b^	139 ± 3.51 ^b^
2 week	269 ± 5.69 ^b^	299 ± 8.21 ^b^	130 ± 6.15 ^a^	174 ± 11.23 ^a^	304 ± 8.42 ^b^	293 ± 5.22 ^b^
3 week	437 ± 6.41 ^c^	454 ± 10.91 ^c^	192 ± 9.78 ^a^	289 ± 12.84 ^b^	470 ± 14.39 ^c^	480 ± 9.32 ^c^
4 week	523 ± 14.24 ^c^	534 ± 11.95 ^c^	282 ± 13.52 ^a^	324 ± 18.42 ^b^	539 ± 10.02 ^c^	577 ± 8.67 ^c^
5 week	728 ± 12.87 ^c^	748 ± 20.03 ^c^	396 ± 15.67 ^a^	454 ± 22.84 ^b^	759 ± 24.00 ^c^	797 ± 14.47 ^c^
1–35 day	2080 ± 24.20 ^c^	2160 ± 43.97 ^c^	1095 ± 42.57 ^a^	1339 ± 49.48 ^b^	2206 ± 47.91 ^c^	2286 ± 28.08 ^c^
Feed consumption, g						
1 week	130 ± 1.33	134 ± 1.33	99 ± 0.11	105 ± 1.59	129 ± 1.33	115 ± 1.59
2 week	358 ± 5.28 ^b^	368 ± 8.55 ^b^	170 ± 5.41 ^a^	180 ± 11.24 ^a^	381 ± 9.78 ^b^	362 ± 7.58 ^b^
3 week	617 ± 7.61 ^c^	616 ± 13.40 ^c^	268 ± 10.86 ^a^	307 ± 17.96 ^b^	633 ± 18.18 ^c^	624 ± 13.76 ^c^
4 week	852 ± 8.97 ^c^	842 ± 13.98 ^c^	415 ± 17.92 ^a^	481 ± 26.86 ^b^	836 ± 11.94 ^c^	886 ± 11.84 ^c^
5 week	1205 ± 18.73 ^c^	1171 ± 36.46 ^c^	635 ± 24.44 ^a^	737 ± 31.17 ^b^	1265 ± 33.61 ^c^	1208 ± 32.54 ^c^
1–35 day	3162 ± 25.64 ^c^	3131 ± 63.22 ^c^	1587 ± 64.95 ^a^	1810 ± 62.29 ^b^	3244 ± 64.09 ^c^	3195 ± 58.97 ^c^
Feed conversion rate (FCR), g of feed/g BWG						
1 week	1.06 ± 0.01	1.07 ± 0.01	1.04 ± 0.02	1.07 ± 0.01	0.96 ± 0.02	0.83 ± 0.02
2 week	1.33 ± 0.01	1.23 ± 0.01	1.31 ± 0.03	1.03 ± 0.01	1.25 ± 0.01	1.24 ± 0.01
3 week	1.41 ± 0.01 ^b^	1.36 ± 0.01 ^b^	1.39 ± 0.03 ^b^	1.06 ± 0.02 ^a^	1.35 ± 0.01 ^b^	1.30 ± 0.01 ^b^
4 week	1.63 ± 0.03	1.58 ± 0.02	1.48 ± 0.02	1.47 ± 0.01	1.55 ± 0.02	1.54 ± 0.02
5 week	1.65 ± 0.01	1.56 ± 0.03	1.60 ± 0.03	1.62 ± 0.03	1.66 ± 0.02	1.52 ± 0.02
1–35 day	1.52 ± 0.01	1.45 ± 0.01	1.44 ± 0.03	1.35 ± 0.01	1.47 ± 0.01	1.39 ± 0.01
Lost birds	0	0	2	1	0	0

^1^ each value represents mean ± SD, the mean initial body weight of chick was 40 g, BWG = the body weight of chick at the end of week − the body weight at the end of preceding week; ^a,b,c^ means in a row marked with various letters are significantly different at *p* < 0.05; ^2^ initial number of birds was 16 (*n* = 16), but in the group 5 AFB_1_ two birds died at 5th week (*n* = 14), in the group 5AFB_1_ + probiotic one bird died at 5th week (*n* = 15).

**Table 2:** Relative weight of liver and kidneys, % EBW ^1,2^.

Organ	Experimental Group					
	1 AFB_1_	1 AFB_1_ + Probiotic	5 AFB_1_	5 AFB_1_ + Probiotic	Control	Control + Probiotic
Liver, % EBW	3.55 ± 0.07 ^b^	2.64 ± 0.09 ^a^	4.57 ± 0.09 ^d^	4.19 ± 0.10 ^c^	2.64 ± 0.11 ^a^	2.60 ± 0.08 ^a^
Kidneys, % EBW	0.97 ± 0.02 ^b^	0.79 ± 0.02 ^a^	1.78 ± 0.08 ^d^	1.56 ± 0.07 ^c^	0.78 ± 0.01 ^a^	0.75 ± 0.02 ^a^

^1^ each value represents mean ± SD, EBW (empty body weight) = body weight before sacrifice − weight of alimentary tract filled with chime; ^a,b,c,d^ means in a row marked with various letters are significantly different at *p* < 0.05; ^2^ Number of birds *n* = 14–16 (see Table 1).

**Table 3:** Concentration of aflatoxin B_1_ in wet excreta (*n* = 14–16, see Table 1), and in the liver and kidney (*n* = 10) at 35th day of age ^1,2^.

Item		Experimental Group			
		1 AFB_1_	1 AFB_1_ + Probiotic	5 AFB_1_	5 AFB_1_ + Probiotic
Excreta, mg/kg	Mean ^1^	0.29 ± 0.10 ^a^	0.89 ± 0.30 ^b^	2.74 ± 0.13 ^c^	4.00 ± 1.61 ^d^
	Range	0.21–0.47	0.52–1.25	2.52–2.88	3.68–7.50
Liver, µg/kg	Mean ^1^	8.86 ± 0.49 ^c^	3.69 ± 0.95 ^a^	11.84 ± 2.99 ^d^	5.88 ± 1.69 ^b^
	Range	8.30–9.64	2.10–4.52	8.42–14.84	3.52–7.20
Kidneys, µg/kg	Mean ^1^	7.93 ± 2.91 ^c^	2.55 ± 0.96 ^a^	13.71 ± 2.76 ^d^	4.06 ± 0.68 ^b^
	Range	4.60–11.62	1.24–3.60	10.10–17.16	2.94–4.62

^1^ Each value represents mean ± SD; ^a,b,c,d^ means in a row marked with different letters differ significantly at *p* < 0.05; ^2^ Number of birds *n* = 14–16 (see Table 1).

**Table 4:** Effects of aflatoxin B_1_ and probiotic supplementation on the histological scores of the liver tissue at 35th day of life ^1^.

Test Group	Vascular Degeneration	Perilobular Inflammation	Intralobular Inflammation	Fatty Changes	Bile Duct Hypertrophy	Fibrosis
**Control**	0.0 ^a^	0.6 ^b^	0.4 ^b^	0.0 ^a^	0.0 ^a^	0.0 ^a^
**Control + probiotic**	0.0 ^a^	0.4 ^b^	0.2 ^b^	0.0 ^a^	0.0 ^a^	0.0 ^a^
**1 AFB_1_**	1.0 ^b^	1.0 ^c^	0.3 ^b^	1.4 ^b^	0.1 ^a^	0.3 ^a^
**1 AFB_1_ + probiotic**	0.0 ^a^	0.0 ^a^	0.0 ^a^	0.0 ^a^	0.0 ^a^	0.0 ^a^
**5 AFB_1_**	1.5 ^c^	2.6 ^d^	2.4 ^c^	1.9 ^c^	1.7 ^b^	2.4 ^b^
**5 AFB_1_ + probiotic**	1.4 ^c^	2.1 ^d^	2.1 ^c^	1.7 ^c^	1.4 ^b^	2.3 ^b^

^1^ Results are expressed as mean of 20 liver sections (2 sections of 10 chickens in each group). Sections with no, mild, moderate, or severe lesions were given scores of 0, 1, 2, and 3, respectively; ^a,b,c,d^ means in a row marked with different letters differ significantly at *p* < 0.05.

**Table 5:** Composition of control diets [g/kg].

Component	Starter	Grower	Finisher
Wheat	348.00	370.00	404.00
Soybean meal	365.00	334.00	299.00
Corn	200.00	200.00	200.00
Limestone	5.50	5.50	10.50
Monocalcium phosphate	13.50	13.00	11.50
NaCl	3.00	3.00	3.00
Soya oil	50.00	60.00	60.00
Vitamin-mineral mixture	10.00	10.00	10.00
L-lys (78%)	1.00	1.50	-
DL-met (98%)	1.00	1.00	-
Feed enzyme ^1^	1.00	1.00	1.00
Wheat starch or probiotic ^2^	2.00	1.00	1.00

^1^ Avizyme 1500 supplied per kg diet: xylanase 1000 U; protease 4000 U and α-amylase 2000 U; ^2^ probiotic preparation contained (per kg): 4.5 × 10^10^ of *Lactobacillus* sp. cells and 4.0 × 10^6^ *Saccharomyces cerevisiae* yeast cells.

**Table 6:** Experimental scheme.

Experimental Group	Dietary Treatment
Control	control diet without additives
Control + probiotic	control diet + probiotic
1 AFB_1_	diet contaminated with 1 mg/kg aflatoxin B_1_
1 AFB_1_ + probiotic	diet contaminated with 1 mg/kg aflatoxin B_1_ + probiotic
5 AFB_1_	diet contaminated with 5 mg/kg aflatoxin B_1_
5 AFB_1_ + probiotic	diet contaminated with 5 mg/kg aflatoxin B_1_ + probiotic

Probiotic preparation was added at 2 g/kg diet between 1st and 14th day of life and at 1 g/kg diet until the end of the experiment.

## Abbreviations

AFB_1_	Aflatoxin B_1_
BWG	Body Weight Gain
ELISA	Enzyme-Linked Immunosorbent Assay
FAO	Food and Agriculture Organization of the United Nations
FCR	Feed Conversion Ratio (kg feed/kg BWG)
H&E	Hematoxylin and eosin
HPLC	High-performance liquid chromatography
LAB	Lactic acid bacteria
EBW	Empty Body Weight
OD	Optic density
